# Who should conduct ethnobotanical studies? Effects of different interviewers in the case of the Chácobo Ethnobotany project, Beni, Bolivia

**DOI:** 10.1186/s13002-018-0210-2

**Published:** 2018-01-26

**Authors:** Narel Y. Paniagua-Zambrana, Rainer W. Bussmann, Robbie E. Hart, Araceli L. Moya-Huanca, Gere Ortiz-Soria, Milton Ortiz-Vaca, David Ortiz-Álvarez, Jorge Soria-Morán, María Soria-Morán, Saúl Chávez, Bertha Chávez-Moreno, Gualberto Chávez-Moreno, Oscar Roca, Erlin Siripi

**Affiliations:** 1 Herbario Nacional de Bolivia, Universidad Mayor de San Andrés, Casilla 10077 Correo Central, La Paz, Bolivia; 2 Museo Nacional de Ciencias Naturales, Calle Ovidio Suarez 26, Cota Cota, La Paz, Bolivia; 3 William L. Brown Center, Missouri Botanical Garden, P.O. Box 299, St. Louis, MO 63166–0299 USA; 4 Instituto Linguistico Chácobo, Beni, Riberalta Bolivia; 5 Comunidad Chácobo de Alto Ivón, Beni, Bolivia; 6 Comunidad Chácobo de Las Limas, Beni, Bolivia; 7 Comunidad Chácobo de Firmeza, Beni, Bolivia; 8 Comunidad Nueva Unión, Beni, Bolivia

## Abstract

**Background:**

That the answers elicited through interviews may be influenced by the knowledge of the interviewer is accepted across disciplines. However, in ethnobotany, there is little evidence to quantitatively assess what impact this effect may have. We use the results of a large study of traditional ecological knowledge (TEK) of plant use of the Chácobo and Pacahuara of Beni, Bolivia, to explore the effects of interviewer identity and knowledge upon the elicited plant species and uses.

**Methods:**

The Chácobo are a Panoan speaking tribe of about 1000 members (300+ adults) in Beni, Bolivia. Researchers have collected anthropological and ethnobotanical data from the Chácobo for more than a century. Here, we present a complete ethnobotanical inventory of the entire adult Chácobo population, with interviews and plant collection conducted directly by Chácobo counterparts, with a focus on the effects caused by external interviewers.

**Results:**

Within this large study, with a unified training for interviewers, we did find that different interviewers did elicit different knowledge sets, that some interviewers were more likely to elicit knowledge similar to their own, and that participants interviewed multiple times often gave information as different as that from two randomly chosen participants.

**Conclusions:**

Despite this, we did not find this effect to be overwhelming—the amount of knowledge an interviewer reported on the research subject had comparatively little effect on the amount of knowledge that interviewer recorded from others, and even those interviewers who tended to elicit similar answers from participants also elicited a large percentage of novel information.

## Background

Quantifying the effects of research methods on results is critical to interpreting those results. This could be particularly true of interview-elicited data, for which qualities of the interviewer may have a large effect. Here, we use results of a large, multi-interviewer dataset on plant uses collected in the Bolivian Amazon to assess interviewer effects and their implications. We found that different interviewers elicited different knowledge patterns that some interviewers systematically elicited knowledge from participants more similar to the interviewer’s own knowledge, and that knowledge reported by one participant to different interviewers was dissimilar. However, all interviewers still elicited much novel information, suggesting that while interview data must be treated with care, it is still a valuable tool in documenting knowledge.

That the results of interviews may be influenced by qualities of the interviewer—his or her gender, age, ethnicity and own attitudes or knowledge—is accepted across disciplines [1–8]. This effect has received more attention in fields that tend to have large number of different interviewers, such as public opinion [2, 4, 8, 9]. In ethnobotany, while the interviewer effect is acknowledged as important [7], it has received relatively attention (with the notable exception of interviewer gender (e.g. [10, 11])). To explore the effects of interviewer identity and knowledge on plant species and uses elicited in interviews, we used the results of a large study of traditional ecological knowledge (TEK) of plant use of the Chácobo and Pacahuara of Beni, Bolivia [12].

Given the availability of previous studies [13–20], the Chácobo are an outstanding possibility to study traditional knowledge over time. In many cases, however, comparisons are difficult to make, given the diversity of investigators and objectives. While a wide variety of studies have examined the efficacy of different methods in ethnobotany (e.g., as summarized in [21]), none have so far have quantitatively focused on the effect of a crucial influence on the results—the individual interviewer. Such effects could come hypothetically through informant selection, with different interviewers accessing different segments of the population, through interviewer effects, with different interviewers eliciting different information even from the same individual, and/or through interviewer bias, with interviewers reporting knowledge sets more similar that which they hold themselves.

Here, we hypothesized (1) that, within a large, contemporary study [12], with a unified training for interviewers, different interviewers would still elicit a different quantity and composition of plants and uses, (2) that these differences would be in particular influenced by the reviewer’s own knowledge of plants and their uses, but that (3) individuals interviewed twice by different interviewers would still report similar knowledge, as interviewer effect or bias would be small compared to differences among individual interviewees.

## Methods

### Study area and ethnobotanical data collection

**Fig. 1:**
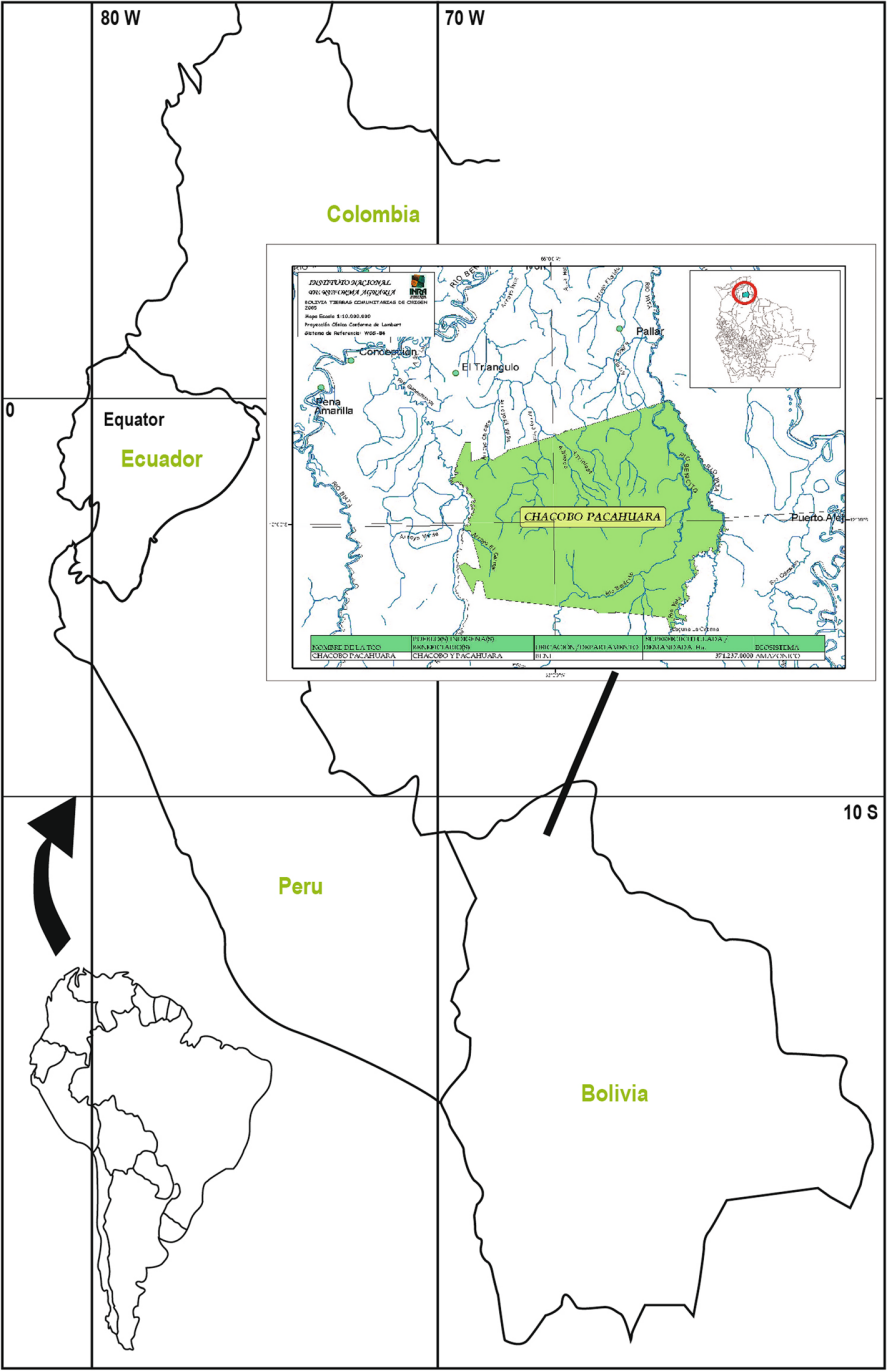
Chácobo territory 2013

The Chácobo and their territory (Fig. 1) have been described in detail in our publication on Chácobo plant-use [12]; thus, we do not repeat details on the study area here.

Our project [12] explored the current traditional knowledge (TK) on plant use of the Chácobo and Pacahuara in Beni, Bolivia, and had three goals: (1) to discover and document current traditional plant knowledge through interviews and surveys, (2) to inventory the current flora of the region, and (3) to repatriate the acquired knowledge as well as previous data to the community.

After obtaining consent from CIRABO (Central Indígena de la Región Amazónica de Bolivia), and before starting fieldwork, we conducted a community meeting in May 2013, involving representatives of all 27 villages in the Chácobo Territory, in order to obtain prior informed consent from all communities. This session included the repatriation of the results of previous studies [20, 21]. In addition, during the project, all available material on Chácobo plant use was translated to Spanish and repatriated [22, 23]. The Chácobo community itself chooses 12 local counterparts to be trained as ethnobotanical interviewers and plant collectors. In September 2013, we conducted a 2-week workshop on ethnobiological methods and plant collection, training the 12 selected counterparts, 10 of which finally acted as interviewers. Training was conducted directly in the field in the central village of Alto Ivón, and involved theoretical exercises (overview on methodology of interviews, collection and herbarium techniques), as well as extensive practical exercises (structuring and testing of questionnaires, test interviews among the participants, field interviews with local community members, plant collection in the field, preparation of herbarium specimens, plant and artifact collection in the local community, data-basing, and initial data analysis).

From November 2013 to May 2015, the 10 Chácobo interviewers collected ethnobotanical information from 301 Chácobo participants (150 women, 151 men, representing almost the entire adult Chácobo population), and over 1500 plant samples were collected. Prior to starting the interviews, every interviewer obtained prior oral informed consent from each participant. Chácobo participants were divided into five age classes (18–30 years old: 58 men, 52 women; 31–40 years old: 31 men, 36 women; 41–50 years old: 35 men, 36 women; 51–60 years old: 15 men, 7 women; and > 60 years old: 12 men, 19 women). Because the study attempted to interview the whole adult Chácobo population, there was originally no emphasis on achieving a balanced age or gender distribution. All interviews were conducted at the homes of the participants by asking participants to freelist their plant knowledge following [24]. All plant uses were categorized following [24]. All interviews were preferably conducted in Chácobo. In a few cases where participants were not fully fluent in Chácobo, interviewers used Spanish as common language. The plant material was collected under permission from the Ministry of Environment and Water of the Plurinational State of Bolivia and was identified and deposited at the National Herbarium of Bolivia (LPB) under the collection numbers of the Chácobo collectors. Nomenclature follows www.TROPICOS.org. Use descriptions were coded after the fact into subcategories and, for some analyses, into six major categories: fodder, fuel, medical, cultural, construction, tool, and food.

All work was carried out following the International Society for Ethnobiology Code of Ethics [25], and under the framework provided by the Nagoya Protocol on Access to Genetic Resources and Fair and equitable sharing of benefits arising from their use of the Convention on Biological Diversity, the Chácobo community retains the copyright of the traditional knowledge of all informants. Any commercial use of any of the information requires prior consensus with informants and communities, and an agreement on the distribution of benefits.

### Data analysis

To examine the extent of interviewer identity on the content of answers elicited, we first asked how the aggregate communities of plants, uses, and unique combinations of plants and use-categories (hereafter “plant-use combinations”) elicited by each interviewer differed. To do this, we ordered informant interviews using non-metric multi-dimensional scaling on distance matrices for plants, uses, and plant-use combinations and tested how well interviewer explained the location of the informant interviews in the resulting ordinations, in comparison to other possible explanatory characteristics, including gender, ethnicity, and age of informant, using the R package vegan [26].

In order to assess whether the interviewer knowledge influenced the quantity of answers elicited, we compared aggregate knowledge sets elicited by each interviewer across interviewers, and for those who were also themselves interviewed, to the knowledge sets they themselves reported, as discussed in depth by [27–30].

To examine how interviewer knowledge influenced the composition of answers, we considered just the subset of data including informants who were interviewed twice, and interviewers who were also themselves interviewed. For each interviewer, we used the locations of informant interviews they had conducted (“interviewer-elicited”), compared to the same informants interviewed by another (“other-elicited”). We considered the average vector from the location of other-elicited interviews to the interviewer-elicited to be a measure of “interviewer effect”. Then, we examined whether interviewers (a) tended to elicit communities of answers more similar to their own answers, by comparing the distance from interviewers own answers to the centroids of the interviewer-elicited answers and of the other-elicited answers, and (b) tended to elicit communities of answers more similar to one another, by comparing the total pairwise distances among interviewer-elicited answers and among other-elicited answers.

To examine how greatly the answers of informants changed in repeat interviews, for each informant who had been interviewed more than once, we compared the ordination location of each interview, quantifying the distance between these two locations as “intra-informant difference”. Such “intra-informant” differences have been elucidated in comparative studies [27–30]. We used the average of these distances, compared to the average of all pairwise distances between interviews “inter-informant difference”, to measure the scale of interviewer effects across the population. To assess the impact of these effects on the overall knowledge base, we calculated the number of unique instances and total mentions for species, uses, and plant-use combinations that were elicited by the interviewer but *not* reported by him or her, as a metric of novel information that elicited, beyond any effect of the interviewer’s own knowledge set.

## Results

**Fig. 2:**
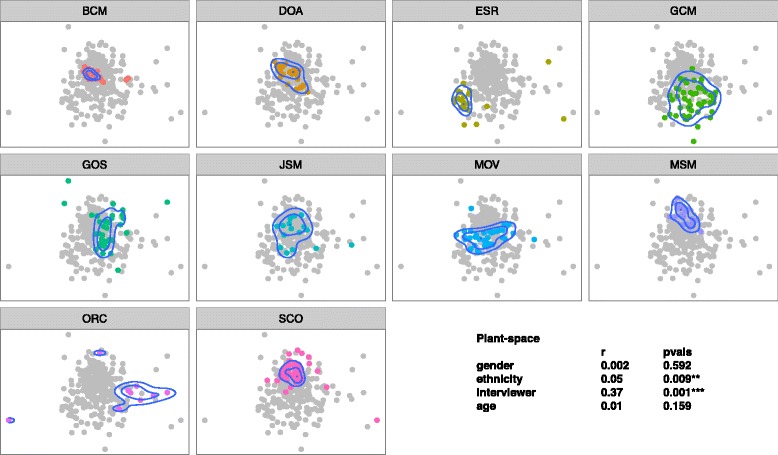
Identity of interviewer (pane title: BCM, DOA, etc.) has a significant effect on the community of plants reported by informants (points in ordination pane) that interviewer was responsible for (points in color). Density contours indicate the greatest concentration of informants

**Fig. 3:**
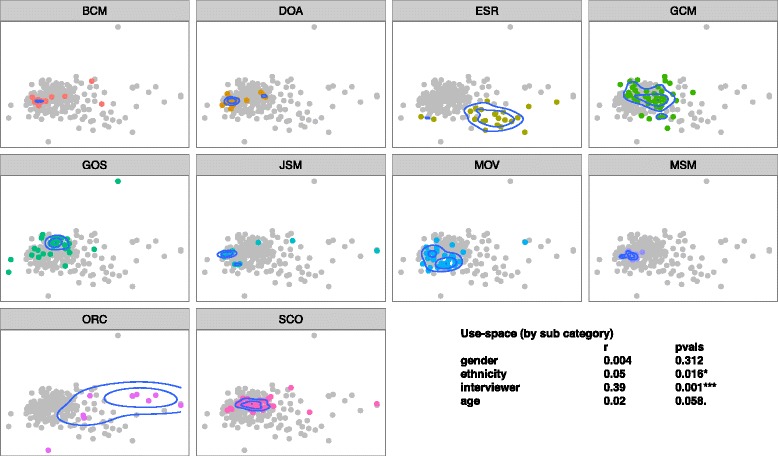
Identity of interviewer (pane title: BCM, DOA, etc.) has a significant effect on the community of uses reported by informants (points in ordination pane) that interviewer was responsible for (points in color). Density contours indicate the greatest concentration of informants

**Fig. 4:**
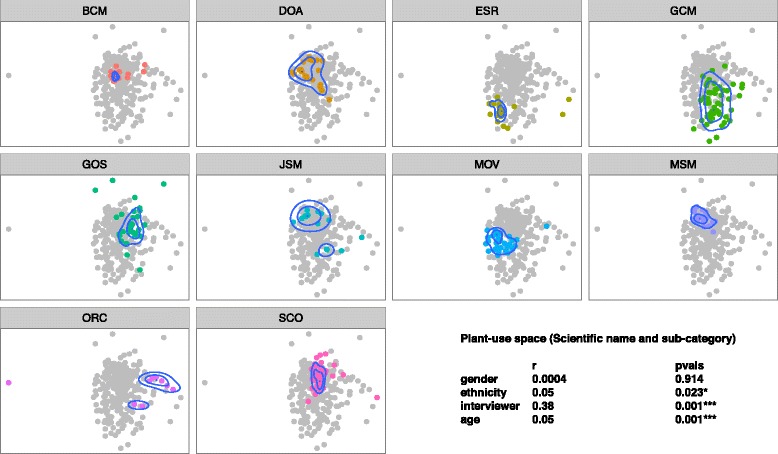
Identity of interviewer (pane title: BCM, DOA, etc.) has a significant effect on the community of plant-use combinations reported by informants (points in ordination pane) that interviewer was responsible for (points in color). Density contours indicate the greatest concentration of informants

In accord with our first hypothesis, we did find that interviewers as individuals elicited distinct communities of plants (Fig. 2), uses (Fig. 3), and plant-use combinations (Fig. 4). Compared to demographic qualities of informants, interviewer identity explained more of the variation in ordination location when fit as an explanatory variable (plants: interviewer *r*^2^ = 0.37, *p* < 0.001, informant ethnicity *r*^2^ = 0.05, *p* = 0.009; uses: interviewer identity *r*^2^ = 0.39, informant ethnicity = 0.05, *p* = 0.016; plant-uses: interviewer identity *r*^2^ = 0.38, *p* < 0.01, informant ethnicity *r*^2^ = 0.05, *p* < 0.001, informant age *r*^2^ = 0.05, *p* < 0.001).

**Fig. 5:**
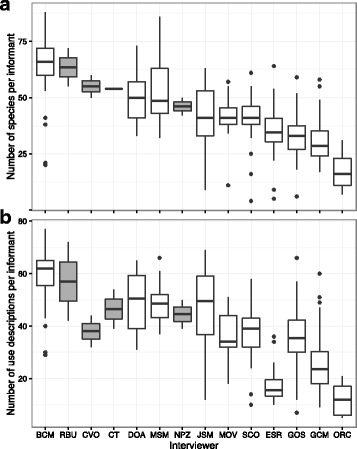
Species (**a**) and uses (**b**) elicited by external interviewers (gray) vs. Chácobo interviewers (white)

**Fig. 6:**
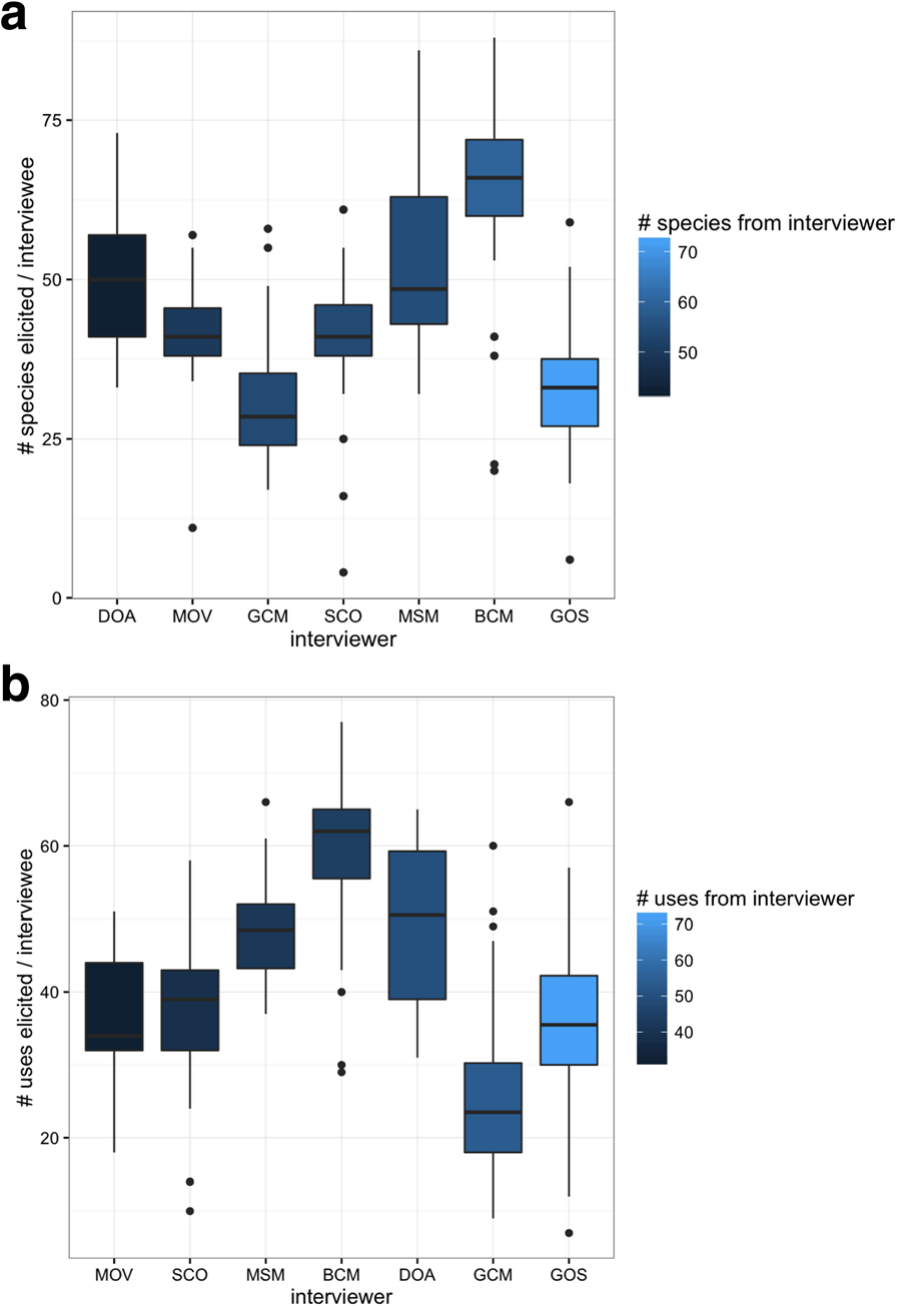
Interviewers who themselves reported more plants (**a**) or uses (**b**) do not tend to elicit more plants or uses from their informants

The number of plants and number of use descriptions elicited from interviewees did differ among interviewers, with some interviewers consistently eliciting many more species (Fig. 5a) and uses (Fig. 5b). Interestingly, the external investigators (RBU, CVO, CT, and NPZ), none of whom spoke Chácobo, nor had personal in-depth knowledge of Chácobo plant knowledge, managed to elicit very similar numbers of species and uses in test interviews during training (Fig. 5). This is in accord with the fact that, for interviewers who were themselves interviewed, the number of species and uses a Chácobo interviewer elucidated from their participants did not relate to their own knowledge. That is, interviewers who themselves had higher knowledge often elicited less information from interviewees than interviewers who held comparatively lower knowledge themselves (Fig. 6).

**Fig. 7:**
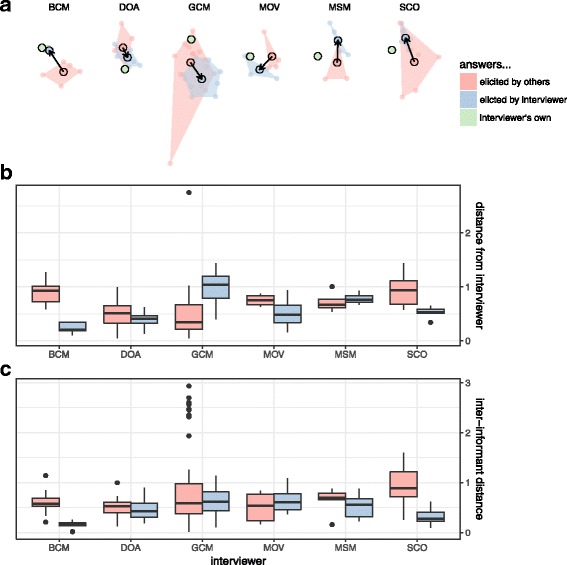
Answers elicited by interviewers about species (red) compared to answers elicited from the same informants when interviews are conducted by another interviewer (blue). A vector arrow compares the centroid of each group (**a**) and the interviewer’s own answers (green). Also shown are the average within each interviewer of the distances of each informant’s answers from their own (**b**) and the average distances among interviews (**c**) for both their own interviews (blue) and those conducted by others (red)

**Fig. 8:**
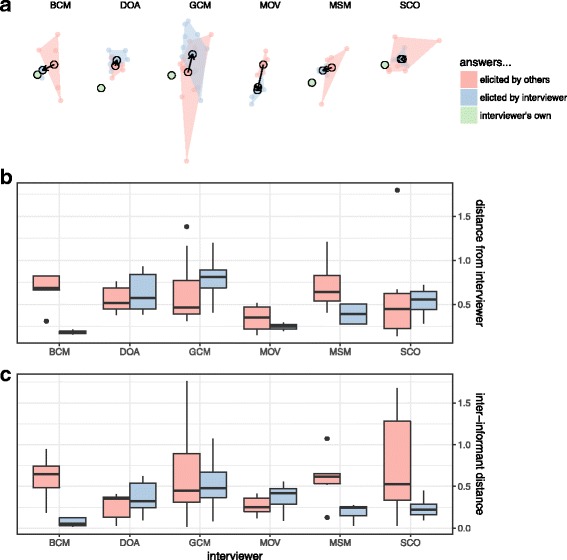
Answers elicited by interviewers about uses (red) compared to answers elicited from the same informants when interviews are conducted by another interviewer (blue). A vector arrow compares the centroid of each group (**a**) and the interviewer’s own answers (green). Also shown are the average within each interviewer of the distances of each informant’s answers from their own (**b**) and the average distances among interviews (**c**) for both their own interviews (blue) and those conducted by others (red)

**Fig. 9:**
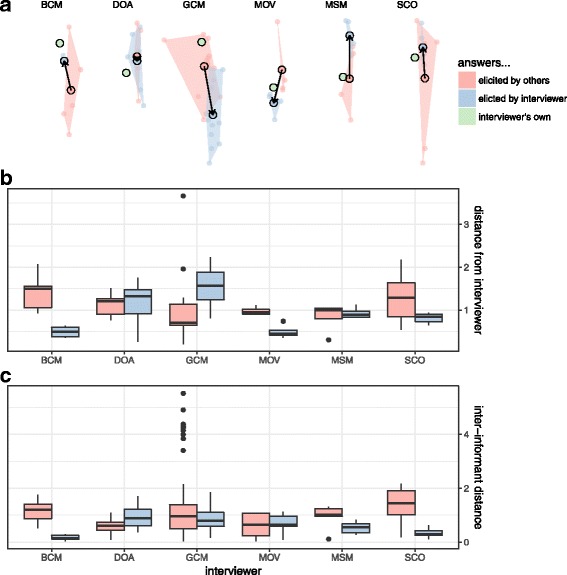
Answers elicited by interviewers about plant-use combinations (red) compared to answers elicited from the same informants when interviews are conducted by another interviewer (blue). A vector arrow compares the centroid of each group (**a**) and the interviewer’s own answers (green). Also shown are the average within each interviewer of the distances of each informant’s answers from their own (**b**) and the average distances among interviews (**c**) for both their own interviews (blue) and those conducted by others (red)

Supporting our second hypothesis, when considering the subset of the population interviewed multiple times by different interviewers, we did see some evidence of interviewer bias. For plant species mentioned, in most cases, interviewers did “pull” answers—elicit answers that were close to their own knowledge sets (Fig. 7a) and “tighten” answers— elicit answers that were closer to one another (Fig. 7b). To some extent, this was also true of uses (Fig. 8) and plant-use combinations (Fig. 9), although in all cases there were great differences among interviewers: some interviewers should more of an effect (BCM, MOV, SCO), while others were equivocal (DOA), or did not show this effect (GCM, MSM).

**Fig. 10:**
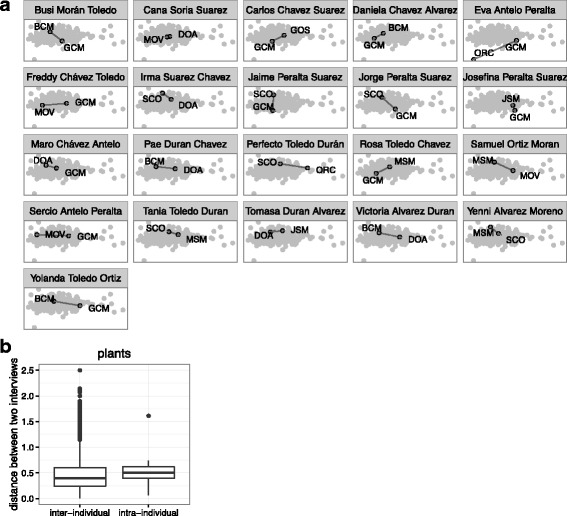
**a** The community of plants reported (black points, labeled with interviewer code) in the two interviews of each informant who was interviewed more than once (pane title), contrasted with all other interviews (gray points). Intra-informant distance represented by a solid black line. **b** The average intra-informant distance compared with the average inter-informant distance

**Fig. 11:**
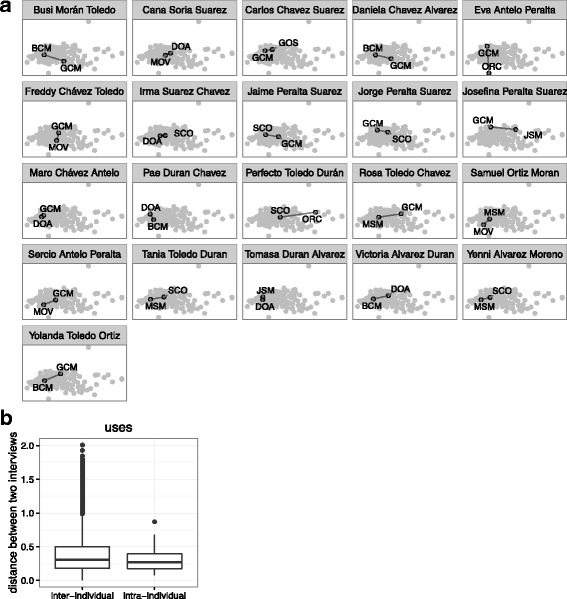
**a** Community of uses reported (black points, labeled with interviewer code) in the two interviews of each informant who was interviewed more than once (pane title), contrasted with all other interviews (gray points). Intra-informant distance represented by a solid black line. **b** Average intra-informant distance compared with the average inter-informant distance

**Fig. 12:**
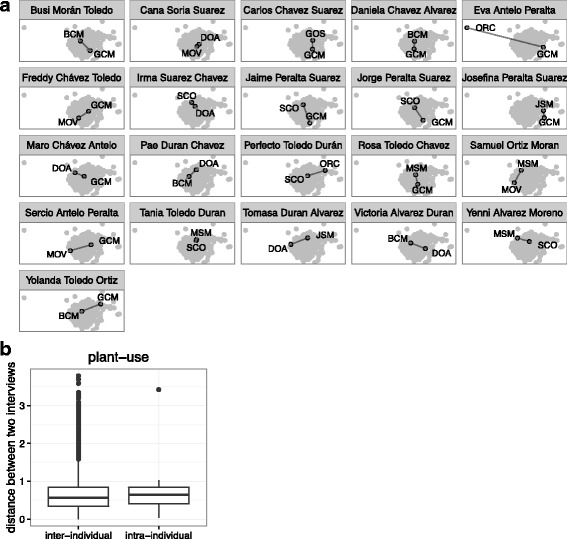
**a** The community of plant-use combinations reported (black points, labeled with interviewer code) in the two interviews of each informant who was interviewed more than once (pane title), contrasted with all other interviews (gray points). Intra-informant distance represented by a solid black line. **b** Average intra-informant distance compared with the average inter-informant distance

Our third hypothesis related to how greatly answers of those interviewed twice would change in repeat interviews. Ordination indicated that, in fact, answers did differ when comparing the distance between repeat interviews of one person (intra-individual) versus all interviews (inter-individual), and in fact, intra-individual distances are not significantly smaller than inter-individual distances, be it for species mentioned (Fig. 10), uses (Fig. 11), or species-use combinations (Fig. 12). This indicates that double interviews of the same person, done by different interviewers, tend to yield such diverse information that they might be counted as separate informants.

Given this clear effect of interviewer influence, did interviewers then still elicit plants, uses, and plant-uses not included in their own knowledge set, or was interviewer influence so strong that interviewers simply had interviewees reiterate their own set of knowledge? In accord with the results showing that the quantity of knowledge elicited from informants does not relate to the quantity known by the interviewer, our data indicate that interviewer bias does not have an overwhelming effect on the composition of knowledge elicited. For interviewers who elicited responses most closely to their own knowledge set (BCM, MOV, and SCO), 60–69% of the plants they elicited were novel (not reported in their own interviews). Similarly, large proportions of uses (55–68%) and plant-use combinations (79–84%) were novel. This was not at all different from the interviewers who elicited the answers least similar to their own knowledge (GCM and MSM) (Table 1). In other words, in every case, each interviewer managed to collect a wealth of new information they themselves did not know about.

**Table 1:** Answers elicited by interviewers in comparison to their own knowledge

Three interviewers who tended to elicit answers similar to their own						
	Species	Species mentions	Uses	Use mentions	Plant-uses	Plant-use mentions
BCM						
Novel	84	1193	27	4059	216	2030
Same as interviewer	57	2866	22	3895	58	2029
Chance novel		29%		51%		50%
MOV						
Novel	102	710	34	2429	282	1372
Same as interviewer	46	1719	16	2181	52	1057
Chance novel		29%		53%		56%
SCO						
Novel	106	1206	29	2903	233	1541
Same as interviewer	48	1697	20	2779	52	1362
Chance novel		42%		51%		53%
Two interviewers who tended to elicit answers more different from their own						
GCM						
Novel	93	686	33	2106	252	1095
Same as interviewer	49	1420	25	2026	60	1011
Chance novel		33%		51%		52%
MSM						
Novel	104	722	29	1783	266	1225
Same as interviewer	49	1061	23	1570	48	558
Chance novel		40%		53%		69%

## Discussion

Many studies have found patterns of the influence of age, or accessibility to markets on traditional knowledge (e.g., [31–39]). The general trend found in relation to the difference in intergenerational knowledge, where young people know more than older people, suggests that this pattern generally could be a result of both knowledge transmission, as well as in situ learning, and be related to the time during which people acquire and use knowledge, with the older informants taking more responsibility in their households, who have a need to learn and apply their knowledge [32, 40–42]. In addition, the trend of a gradual decrease in knowledge from the old to the very young has been related to increased exposure of the new generations to new technology and services (e.g., pharmacies, paramedics, clinics, and different types of building materials, tools, and alternative utensils available in commercial centers) [35, 36, 38, 43, 44]. Some studies confirmed the hypothesis that people who are relatively isolated from the market economy share more traditional knowledge than people who live close to cities or larger towns [32]. Researchers have postulated that some resources may no longer be used due to industrialization or acquisition of technology services such as changes from wood to gas stoves, and a replacement of medicinal plants by modern medicine [45, 46].

General issues of interview methodology have been discussed ad infinitum in standard cognitive social science literature (e.g., [46–65]. In addition, large numbers of studies have discussed issues like interview structure and content, as well as the heterogeneity of interviews [66], or the number of interviews needed for representative studies, as well as the great complexity of interview situations, and the influence of researcher’s experience [67]. Even issues like the influence of researcher confidence [68], the number of researchers in a team [67], resourcing [69], interview saturation [70], and informant selection [71] have been broadly discussed.

However, no study has so far considered the influence of individual interviewers on results obtained from large groups of participants in an indigenous setting, although such effects are well known from other fields of research, e.g., public health [3], and especially marketing [1], and their influence on statistical model has been widely discussed [8, 9]. Our study supported our hypotheses that different interviewers elicit different sets of knowledge and that interviewers are more likely to elicit knowledge similar to their own. In fact, and contrary to our expectations, participants interviewed multiple times often gave as different information as any two randomly chosen participants would have given. Despite this, we did not find this effect to be overwhelming—the amount of knowledge of an interviewer on the research subject had comparatively little effect on the amount of knowledge yielded, and even the interviewers who tended to elicit the most similar answers from their informants to their own knowledge also elicited a large percentage of novel information.

The fact that the scientific trainers were able to elucidate similar quantities of knowledge levels local interviewers suggests the corollary: with appropriate support, local interviewers can yield similar results to those of external investigators. Given the unique sets of knowledge available to individuals, and the time constraints that usually exist for external investigators, it is apparent that multiple, local interviewers will allow ethnobotanical studies to most comprehensively document knowledge.

## Conclusions

We suggest that the training of indigenous interviewers and plant collectors should be seriously considered for conducting any studies involving the documentation of traditional knowledge. Our results indicate that, at least in case of the Chácobo, the interviewer effect was negligible, given the very large number of participants interviewed. We suggest that the combination of indigenous interviewers, and a very large set of participants, is an excellent strategy to elucidate a maximum of information in ethnobotanical studies.

As final result of our project, all collected knowledge was repatriated to the Chácobo in a plant guide [72].

## Abbreviations

BCM: Bertha Chávez Moreno
CIRABO: Central Indígena de la Región Amazónica de Bolivia
CT: Carolina Tellez
CVO: Carlos Vega Ocaña
DOA: David Ortiz Álvarez
ESR: Erlin Siripi
GCM: Gualberto Chávez Moreno
GOS: Gere Ortiz Soria
JSM: Jorge Soria Morán
MOV: Milton Ortiz Vaca
MSM: María Soria Morán
NPZ: Narel Y. Paniagua Zambrana
ORC: Oscar Roca
RBU: Rainer W. Bussmann
SCO: Saúl Chávez
TCO: Tierra Comunitaria de Origen (Communal lands of original inhabitants)
TEK: Traditional Ecological Knowledge
TK: Traditional Knowledge
